# Supplementary material for: Complexome profiling of the Chlamydomonas psb28 mutant reveals TEF5 as an early PSII assembly factor
Source: Plant Cell. 2025 Mar 17;37(6):koaf055. doi: 10.1093/plcell/koaf055 (PMC12164590; doi:10.1093/plcell/koaf055)
Supplement: koaf055_Supplementary_Data [file koaf055_supplementary_data.zip › psb28_interactive.zip!/plots/intensity/qualityPlots/peptides/MPC1_Cre06.g286250.t1.2.html]

### Cre06.g286250.t1.2

Trivial name: MPC1  
SeqCoverage [median]: 26.90  
Mass: 32.08 kDa  
  
 

| Sequence | MissedCleavage | Mass [Da] | wtIntensity | muIntensity | Score |
| --- | --- | --- | --- | --- | --- |
| ALLQSGGGK | 0 | 829.47 | 2814.68 | 0.00 | 91.85 |
| AYGASVGR | 0 | 779.39 | 754.49 | 742.27 | 79.47 |
| GAITAIR | 0 | 700.42 | 510.76 | 663.86 | 106.76 |
| GMYAGYGAFMLR | 0 | 1335.61 | 828.37 | 1205.41 | 88.95 |
| LMTQGASGR | 0 | 919.45 | 2924.82 | 0.00 | 90.15 |
| LQAMIGGGGLK | 0 | 1043.58 | 0.00 | 2828.01 | 87.48 |
| LQTGEFK | 0 | 821.43 | 345.69 | 2024.03 | 104.45 |
| VPTEVVK | 0 | 770.45 | 2973.04 | 0.00 | 128.48 |
| YKNLFDATVTIAR | 1 | 1510.81 | 695.60 | 3366.34 | 75.59 |
|  |  |  | 11847.5 | 10829.9 |  |
